# Insights into Hypermetallic Molecules En Route to Multiple Optical Cycling Centers: Thermodynamic and Spectroscopic Trends in Mg and Ca Bearing Acetylides

**DOI:** 10.1021/acs.jpca.5c03484

**Published:** 2025-07-08

**Authors:** Ella Brudner, Tomer Gur, Nadav Genossar-Dan, P. Bryan Changala, Michael C. McCarthy, John F. Stanton, Joshua H. Baraban

**Affiliations:** † Department of Chemistry, 26732 Ben-Gurion University of the Negev, Beer Sheva 8410501, Israel; ‡ 61814 Center for Astrophysics—Harvard & Smithsonian, Cambridge, Massachusetts 02138, United States; § Quantum Theory Project, Departments of Chemistry and Physics, 3463 University of Florida, Gainesville, Florida 32611, United States

## Abstract

Hypermetallic molecules have been proposed as novel candidates for laser cooling and precision physics measurements, but species such as CaCCMg have yet to be synthesized and detected, despite recent successes in characterizing other related species of fundamental and astrochemical interest. Here, we examine the thermodynamics of the relevant alkaline earth metal chemistry with an acetylene precursor via ab initio electronic structure calculations. Experimental measurements of optical emission from a discharge-assisted laser ablation expansion source highlight the potential importance of electronically excited atoms in producing the desired species.

## Graphical abstract

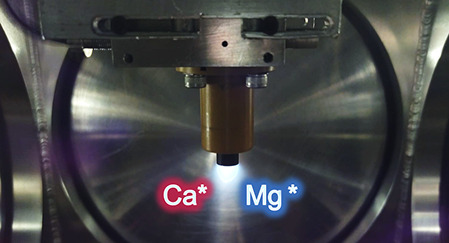

## Introduction

Metal-bearing molecules with increasing complexity and versatility are of interest in quantum technologies, precision physics, and ultracold chemistry. The potential for laser cooling, i.e., the availability of a closed, efficient excitation–emission cycle, is a key characteristic in selecting new candidates for research in these fields. By analogy to the well-established laser cooling of atoms,^1–4^ suitability for cooling in molecules is often achieved by the presence of an atom with an isolated unpaired electron, termed an “optical cycling center” (OCC).^5–9^ The criteria for successful cooling amid the complexity of molecules can be summarized by three conditions:^10^ first, the molecule must have strong one-photon transitions to enable sufficiently rapid repeated interactions with the cooling laser photons, typically requiring an electronic band system; second, said band system should possess highly diagonal Franck–Condon factors (FCF), such that emission from the excited state returns essentially exclusively to the ground state; third, there should be no other decay channels that will lead to population loss from the two-level cycling system. The pursuit of OCCs in increasingly large and varied molecules is thus an important but challenging goal.

Recently it was proposed that multiple OCCs in a single molecule might provide a novel direction forward. Multiple OCCs may increase the rate of photon scattering^11^ or the versatility of cold molecule manipulation by cooling using one of the OCCs and conducting precise measurements with the other.^12^ Due to these significant potential applications, identifying candidate polyatomic molecular motifs for multiple OCCs has attracted considerable interest, including alkaline earth metals (Mg, Ca, Sr, etc.) attached to electron-acceptor ligands such as acetylides.^13,14^

The favorable OCC characteristics of alkaline earth metal-bearing molecules can be understood by recalling that an OCC is usually designed for “atom-like” hybridization of the molecular orbitals, thus reproducing the simple atomic level structure and its closed excitation–emission cycle. The M^+^L^–^ bond between the alkaline earth atom and the electron-withdrawing group creates an *sp* hybridized orbital on the metal atom that is occupied by an unpaired electron, which interacts only weakly with the rest of the molecule. This electronic structure leads to highly diagonal FCF for transitions involving the electron localized on the metal atom.^15,16^

Accordingly, it is conceivable that two metal atoms on the same acetylide linker (M–CC–M*′*) can form two separate OCCs, if they do not interact too strongly. It is further possible to extend the (−CC−) units in the chain length,^17–19^ allowing for the examination and tuning of the critical unpaired electron spin density as a function of distance.^16^

Several spectroscopic observables can report on interactions between two potential OCCs in the same molecule. One diagnostic is the change in the metal–ligand bond length or ligand geometry relative to a species containing a single OCC.^16^ Perhaps more fundamentally, small singlet–triplet gaps in the low-lying states will indicate noninteracting metal centers. Ultimately, a full complement of spectroscopic properties (geometries, vibrational frequencies, and (transition) dipole moments) are needed to understand the structure–function relationships linking molecular composition and electronic structure with the optical properties favorable for photon cycling. Theoretical predictions of these molecular quantities as well as their thermochemistry will assist in the synthesis, detection, and applications of these species.

The formation and spectroscopic properties of M–CC–M*′* and related organometallics are also of interest in astrochemistry,^20–22^ under extreme conditions where such species are known to form.^23^ Changala et al. recently measured the laboratory spectrum of MgC_2_, which led to its detection in a circumstellar envelope,^24^ followed closely by CaC_2_.^25^ Although these species are likely formed under kinetic control in both these astrophysical and laboratory conditions, their thermochemistry is an essential starting point for considering both their potential astrophysical relevance and gas-phase synthesis routes in the laboratory.

In this work we discuss the spectroscopy and thermochemistry of M–CC–M*′* alkaline earth metal (Mg, Ca) acetylides and other related molecular species, along with reaction mechanisms for their formation. High accuracy ab initio calculations based on (equation of motion) coupled cluster methods provide a reliable and comprehensive picture of the relevant properties, with an eye toward laser cooling applications. We also present experimental evidence for the involvement of excited metal atoms in the laboratory discharge-assisted laser ablation synthesis of these organometallic species.

## Methods

### Computational Methods

Ab initio calculations of molecular properties were performed using CFOUR.^26,27^ Optimized geometries were obtained with all-electron (AE) coupled cluster calculations at the CCSD­(T)^28^ level of theory with the Dunning correlation-consistent core–valence basis set cc-pCVQZ.^29–31^ Unrestricted Hartree–Fock (UHF) reference determinants were used for open-shell species. For Ca the effective core potential (ECP) ECP10MDF was employed to account for the first 10 electrons, and for Mg the 1*s* orbitals were left uncorrelated.^32^ Harmonic vibrational frequencies and zero point energies (ZPE) were obtained by numerical differentiation of analytic gradients. For symmetric bimetallic molecules, whose multireference diradical singlet ground states are an inappropriate target for standard CC methods, EOM-DIP-CCSD/cc-pCVQZ calculations^33^ were used to access the ground state via a single closed-shell dianion reference.

### Experimental Methods

Optical emission spectroscopy was used to characterize the visible plasma glow from a discharge-assisted^34^ laser ablation^15^ supersonic jet source nearly identical to that used in recent studies of alkaline earth acetylides.^16,17^

A gas pulse of pure argon (UHP 99.999%) expanded into a large vacuum chamber via a pulsed valve (General Valve, Series 9) with an opening time of 350–500 μs at a repetition rate of 20 Hz. A rotating metal rod (Mg, Ca) was ablated by the focused second harmonic of an Nd:YAG laser (Innolas SpitLight 1200; 10 ns, 50 mJ) at the onset of the gas pulse. The entrained ablation products then passed through a nozzle extension containing two copper ring electrodes separated by 13 mm with a pulsed bias potential of 1 kV. Spontaneous emission from the discharge following expansion into vacuum was collected into an IsoPlane 320 spectrograph equipped with a holographic diffraction grating of 1200 lines/mm, coupled to a PI-MAX4 1024i ICCD camera (Princeton Instruments). Data analysis was performed using MATLAB,^35^ and the results were compared to the NIST atomic spectra database.^36–40^

## Results and Discussion

### Reaction Network and Thermochemistry

Schematic overviews of the thermochemistry of 2Mg, 2Ca, and Ca + Mg with acetylene are shown in Figures 1, 2, and 3, respectively. Note that while ground-state reactants appear to lead exothermically only to diatomic (and presumably cluster^41^ products), low-lying electronic excitations of the alkaline earth metal atoms (the lowest singlet states are denoted in blue (Mg) and red (Ca) in the energy diagrams) transform the thermochemical picture entirely^42–44^ and potentially open downhill pathways to all considered products; we have observed emission from such excited states in laboratory discharge-assisted laser ablation (Figure 4). It appears that the discharge-enhanced production of various organometallics in such sources may be due to this excitation mechanism as opposed to or in conjunction with breakdown of metal clusters.

**Figure 1:**
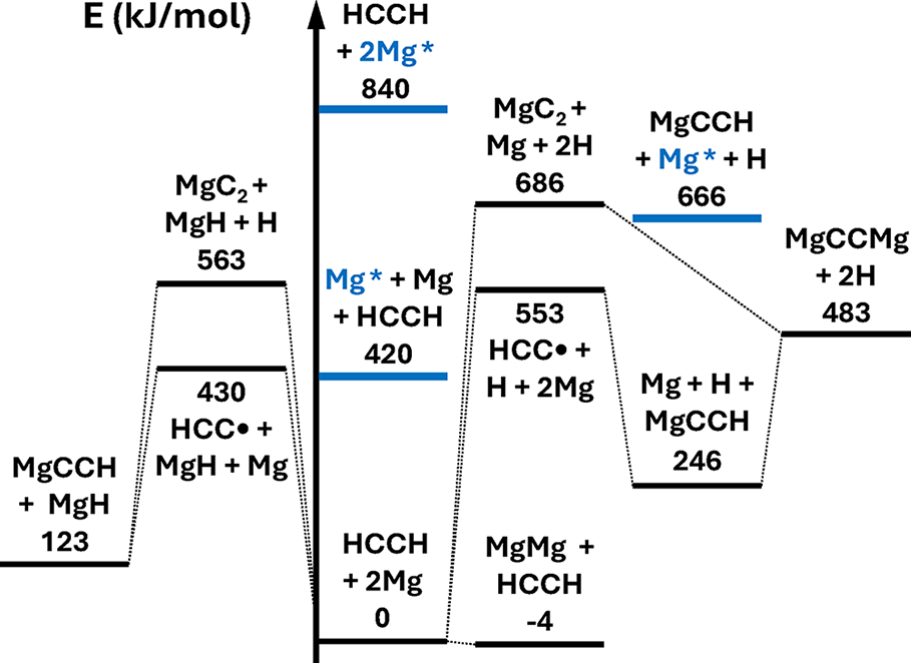
Energy diagram for Mg and acetylene chemistry. Excited states of Mg are marked in blue to highlight their potentially significant effect on the reaction energetics, which include zero-point energies.

**Figure 2:**
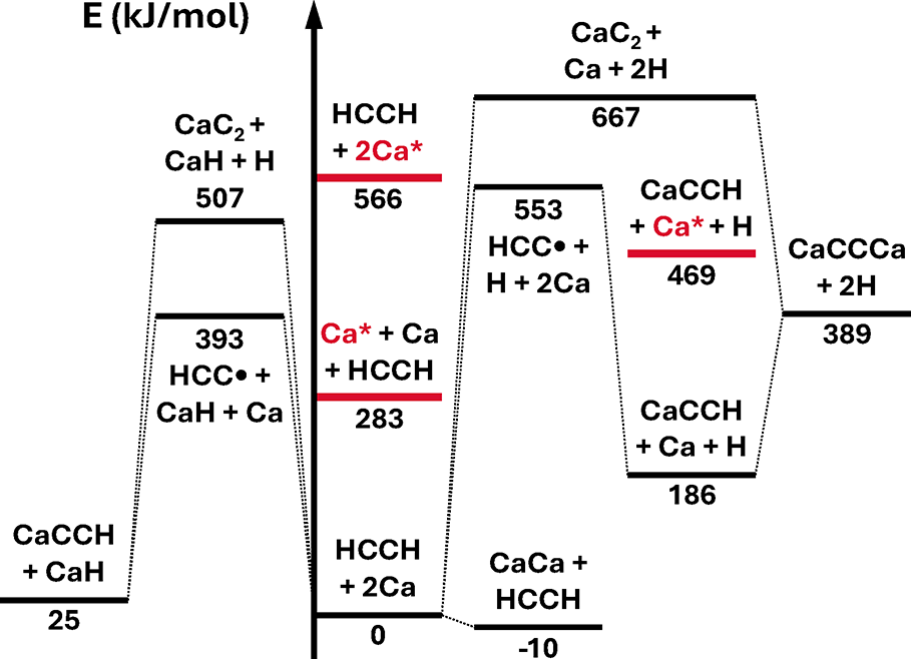
Energy diagram for Ca and acetylene chemistry. Excited states of Ca are marked in red to highlight their potentially significant effect (albeit less so than with Mg) on the reaction energetics, which include zero-point energies.

**Figure 3:**
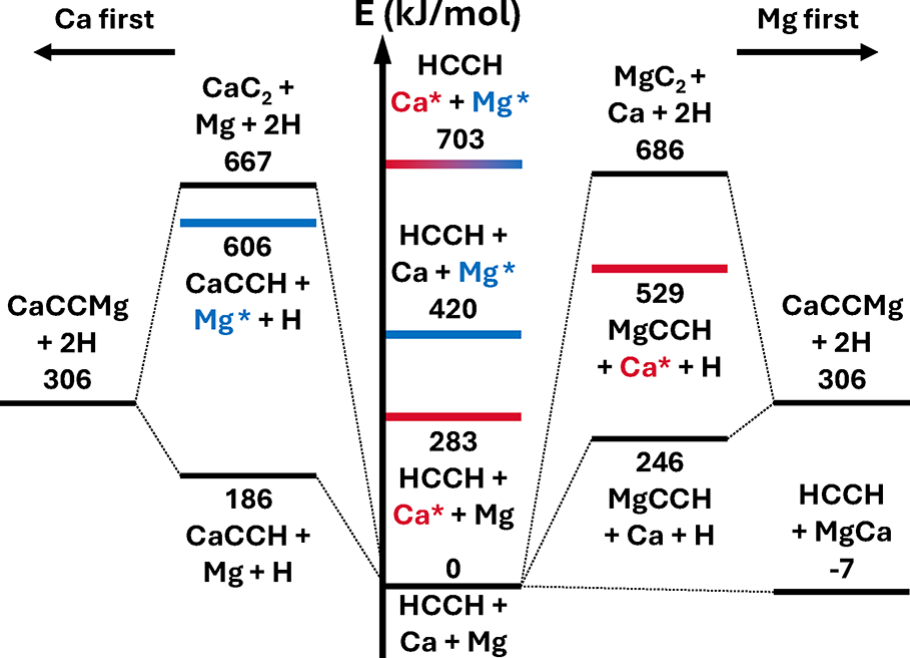
Energy diagram of Ca and Mg chemistry with acetylene. Excited states of Mg are marked in blue and excited states of Ca in red to highlight their potentially significant effect on the reaction energetics, which include zero-point energies.

**Figure 4:**
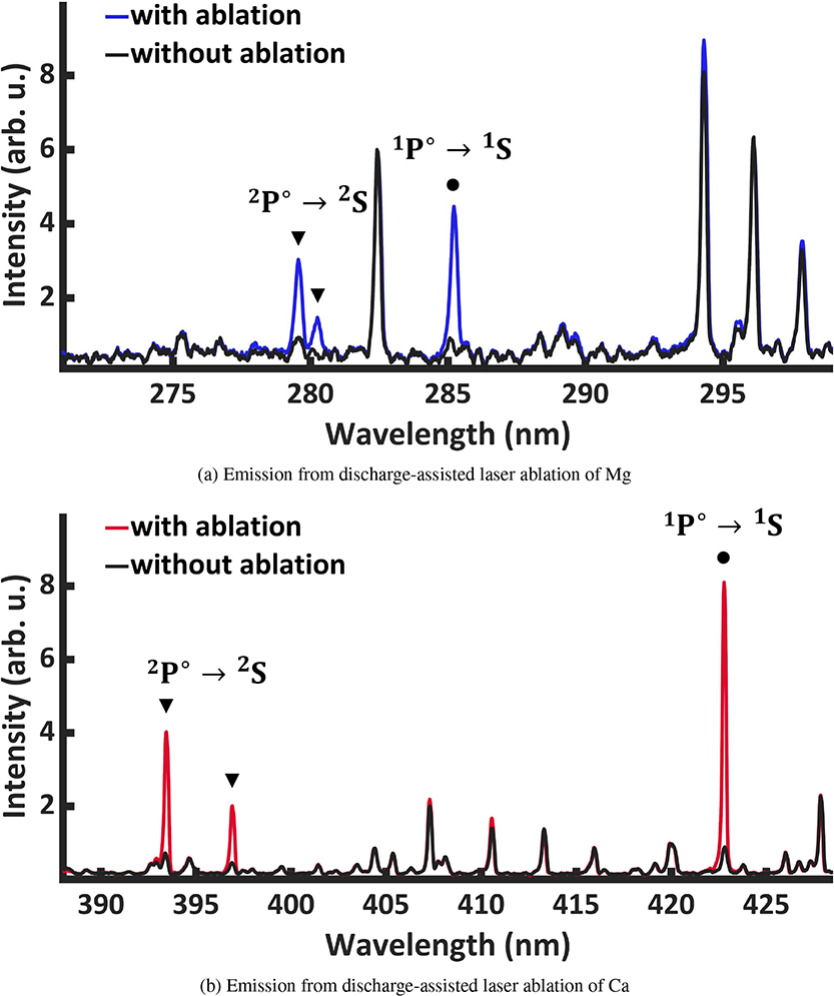
Optical emission spectra of the plasma jet generated by our discharge-assisted laser ablation source with (a) Mg and (b) Ca rod targets ablated in Ar carrier gas en route to the discharge. The observed emission lines correspond to the electronic transition from the 3p to 3s orbital in Mg (or Mg^+^), or 4p to 4s in Ca (or Ca^+^), respectively. The specific transitions shown are from the ^1^P° state to the ^1^S ground state of the neutral atom (marked by •), and the analogous cation transition (split into two due to spin–orbit coupling) from the ^2^P_1/2_^°^ and ^2^P_3/2_^°^ levels to the ^2^S cation ground state (marked by ▼). No emission is observed without the discharge (not shown), and the lines observed both with and without ablation are due to the Ar carrier gas.

In the presence of both the ablation laser and the electric discharge, we detected emission lines from excited states of neutral metal atoms and cations,^36–40^ apparently populated by the electric discharge, since no emission was observed with ablation alone (Figure 4) with our source geometry. For both Mg and Ca metals we clearly observe the lowest-lying singlet state of the neutral and the lowest-lying doublet state of the cation (^1^P° → ^1^S and ^2^P° → ^2^S respectively). This observation alone could provide a reasonable explanation for similar experiments demonstrating that electric discharge increases the production of organometallic molecules by up to a factor of 5.^16^ Transitions related to higher states were not observed, and other transitions involving these states either fall outside our detection range or are expected to be weak, such that the measurements reported here constitute a self-consistent set. Nevertheless, we tentatively assigned a very weak emission line originating from a triplet state (^3^D → ^3^P°) for Mg, and in general, we caution that it is still conceivable that other excited levels might be present in the discharge ablation source.

In the following we discuss the various species in the reaction network, grouped by type.

### M–X

The simplest molecules encountered belong to the diatomic category M–X (X = H, Mg, Ca). Energetically, formation of the bimetallic diatomics is the most favorable out of all the possibilities considered in the reaction networks shown, resulting from a single exothermic step. Such bimetallic diatomic species have been synthesized and characterized spectroscopically,^45–47^ and it is likely that this pathway continues rapidly to M_*n*_ (*n* ≥ 3) clusters.^41^ The M–H forms are both higher in energy and involve a radical chain reaction or direct substitution. Nevertheless, these molecules have been obtained by laser ablation.^48,49^

The bimetallic diatomic species are important here primarily due to their thermodynamic stability, because the electronic structure in the directly bonded M_2_ and M–M*′* species is not comparable to that of bimetallic acetylides. The oxidation state of the metal atoms is different and overall the electronic structure of the alkaline earth dimers is somewhat unusual because the bond forms between two closed-shell species; accordingly, while the ground state is singlet, its bond order is formally zero, and the higher-lying triplet state is much more strongly bound.^45^ The singlet–triplet gap is consistently greater than 1 eV (1.78044 eV for Mg_2_, 1.01829 eV for Ca_2_, and 1.22388 eV for MgCa; or equivalently 172 kJ/mol for Mg_2_, 98 kJ/mol for Ca_2_, and 118 kJ/mol for MgCa) making formation of the triplets significantly endothermic without electronic excitation of the reactants. The large difference indicates unsurprisingly that the two metal centers interact strongly, since the two metal atoms share a bond whose character changes qualitatively with multiplicity.

The extent of the interaction between the metal atoms is also reflected in the singlet vs triplet variation of other properties such as the geometry, dipole moment (for MgCa) and vibrational frequencies (Tables S1, S6, and S7). The bond length in the bimetallic M–M*′* molecules changes significantly between the singlet and triplet states, decreasing by roughly 1 Å. The dipole moment of MgCa increases dramatically from singlet to triplet, presumably as the increased bonding redistributes electron density. The singlet vibrations are similarly significantly lower than in the triplets and remarkably low in an absolute sense, again reflecting the zero bond order.

A more appropriate electronic comparison for the bimetallic acetylides is to the family of M–O–M species.^50^ According to Ostojić et al.,^51,52^ the electronic structure prediction for Mg–O–Mg and Ca–O–Ca shows dominant ionic M^+^–O^2–^ character. Both are predicted to be linear in the ground singlet state ^1^Σ_g_^+^ and in the first excited triplet state ^3^Σ_u_^+^. Furthermore, the calculated singlet–triplet gap in both molecules is very small (<0.0831 eV in Mg–O–Mg and <0.0479 eV in Ca–O–Ca) suggesting that the two unpaired electrons do not interact strongly. The similar geometries (<0.004 Å change in the Ca–O bond length and even less for the Mg–O bond), electric dipole moment functions, and vibrational frequencies of the singlet and triplet states point to similar electronic environments with the two multiplicities and support the picture of two weakly interacting metal atoms.^51,52^

### Cyclic MC_2_

Cyclic MC_2_ species have long been thought more stable than the linear MCC forms,^42,53,54^ but have only been detected recently in IRC+10216 and the laboratory.^24,25^ Since these species are relatively high energy, as shown in Figure 3, this implies that it should be possible to form the much lower-energy M–CC–M*′* molecules under similar conditions. The structural similarity of the linear MCC species with the analogous MCCH, generally and relative to the MC_2_ cyclic isomers, remains consistent with the isolation of the metal atom from the linker.^16^

### (Bi)­metallic Acetylides

The calculations performed in this work support both the feasibility of producing M–CC–M*′* species by discharge-assisted laser ablation and their potential suitability for optical cooling, as manifested in the thermodynamic data and comparison to the M–O–M*′* trends as described below.

Despite the endothermic nature of the metal atom substitutions, these reactions with acetylene are known to yield metal acetylides and even much higher energy species,^21,24,25,55,56^ especially with the discharge enhancement by cluster breakdown and/or atomic excitations, which in some cases make the reactions exothermic. Nevertheless, the M-CC-M molecules have yet to be detected, even though for example CaCCMg possesses a dipole moment comparable to that of the readily detected MgCCH. Other aspects of the discharge and ablation chemistry conditions, perhaps related to the necessary double substitution, are presumably responsible and may provide the key(s) to successful synthesis.

Due to the variety of structures reported in the literature for potentially analogous Be compounds,^57–59^ we also conducted a low-level survey for other Mg_2_C_2_ isomers that might compete with the formation of the linear bimetallic species. Only one potential kite-shaped isomer was found some tens of kJ/mol higher, but upon optimization at a higher level of theory it appears to be a transition state.

Theoretical support for M–CC–M species as promising candidates for laser cooling has been reported previously^12,13^ and our results are also consistent with noninteraction of the two metal centers. Primary indicators are the decreased singlet–triplet gap compared to M–O–M species, alongside the insensitivity of geometry^13,14^ and dipole moments to spin multiplicity. Furthermore, the similar singlet and triplet vibrational frequencies of M–CC–M species suggest that the weak diradical interactions are not strongly enhanced at deformed geometries. Altogether, these data point to a robust ionic character of the metal–linker bond that bodes well for two isolated metal atoms.

## Conclusions and Further Work

Thermodynamic aspects of the alkaline earth metal reaction network with acetylene imply that it should be possible to generate bimetallic species, especially in the presence of excited atoms. The ground-state properties of these molecules calculated here using methods that achieve good agreement with experiment for related species indicate that detection of the polar asymmetric species by rotational spectroscopy should be achievable if the production challenge can be overcome. They also continue to support the hypothesis that the two metal atoms can constitute isolated optical cycling centers, but excited state calculations are needed to explicitly investigate the relevant spectroscopic characteristics and guide future experiments that would be prerequisites to laser cooling.

## Supplementary Material



## Notes

Tables of molecular geometries, rotational constants, dipole moments, vibrational frequencies, and singlet–triplet gaps (PDF)
