# Supplementary material for: Insights into Hypermetallic Molecules En Route to Multiple Optical Cycling Centers: Thermodynamic and Spectroscopic Trends in Mg and Ca Bearing Acetylides
Source: J Phys Chem A. 2025 Jul 8;129(28):6385–90. doi: 10.1021/acs.jpca.5c03484 (PMC12278219; doi:10.1021/acs.jpca.5c03484)
Supplement: Supplementary file 1 [file jp5c03484_si_001.pdf]

Supporting Information for:

Insights into Hypermetallic Molecules en route to Multiple Optical Cycling Centers: Thermodynamic and Spectroscopic Trends in Mg and Ca Bearing Acetylides

Ella Brudner,[1, a)] Tomer Gur,[1, a)] Nadav Genossar-Dan,[1] P. Bryan Changala,[2] Michael C. McCarthy,[2] John F. Stanton,[3] and Joshua H. Baraban[1]

[1)] *Department of Chemistry, Ben-Gurion University of the Negev, Beer Sheva 8410501, Israel*

[2)] *Center for Astrophysics | Harvard & Smithsonian , Cambridge, MA 02138, USA*

[3)] *Quantum Theory Project, Departments of Chemistry and Physics, University of Florida, Gainesville, Florida 32611, USA*

(*Electronic mail: jbaraban@bgu.ac.il)

(Dated: 26 June 2025)

[a)] E. B. and T. G. contributed equally.

I. MOLECULAR GEOMETRIES

Bond lengths are given in Å, angles in degrees.

TABLE S1. Calculated diatomic bond lengths in Å. Note that for each of the M-M′ molecules the bond length for the triplet is shorter than for the singlet, due to the different bond order with the different multiplicities.

Molecule	r(M-H)[Å]	
MgH	1.7295	–
CaH	2.0037	–
	Singlet r(M-M′) [Å]	Triplet r(M-M′) [Å]
MgCa	4.1023	3.1109
MgMg	3.9464	2.9864
CaCa	4.3421	3.4654

TABLE S2. Triatomic molecular geometries.

	r(MC) [Å]	r(CC) [Å]	
Linear MgCC	2.0194	1.2310	–
Linear CaCC	2.5968	1.2141	–
	r(M$\cdots$CC) [Å]	r(CC) [Å]	$\angle$(CMC)°
Cyclic MgC_2	1.9107	1.2717	71.59
Cyclic CaC_2	2.1126	1.2693	73.28

TABLE S3. Calculated M-CC-X molecular geometries in Å. Note that the M-C bond lengths do not vary significantly, implying that the bonds are largely separable in the bimetallic species.

	r(MC) [Å]	r(CC) [Å]	r(CX) [Å]
HCCH	1.0621	1.2037	1.0621
MgCCH	2.0393	1.2228	1.0650
CaCCH	2.3156	1.2282	1.0656
^{1}MgCCMg	2.0381	1.2435	2.0381
^{3}MgCCMg	2.0356	1.2407	2.0356
^{1}CaCCCa	2.3090	1.2509	2.3090
^{3}CaCCCa	2.3015	1.2499	2.3015
^{1}MgCCCa	2.0313	1.2446	2.3096
^{3}MgCCCa	2.0308	1.2450	2.3090

II. ROTATIONAL CONSTANTS

TABLE S4. Calculated rotational constants for linear molecules.

Molecule	B [MHz]
MgH	174625.42
CaH	128050.31
Linear MgC_2	5445.82
Linear CaC_2	3105.07
MgCCH	4947.39
CaCCH	3369.70
^{1}MgCa	2003.56
^{1}MgMg	2705.76
^{1}CaCa	1341.48
^{3}MgCa	3483.92
^{3}MgMg	4724.98
^{3}CaCa	2106.02
^{1}MgCCCa	1033.25
^{1}MgCCMg	1453.96
^{1}CaCCCa	726.98
^{3}MgCCCa	1033.44
^{3}MgCCMg	1453.82
^{3}CaCCCa	728.32

TABLE S5. Calculated rotational constants for cyclic molecules.

Molecule	A [MHz]	B [MHz]	C [MHz]
Cyclic MgC_2	9359.67	11399.12	52314.19
Cyclic CaC_2	6598.24	7551.29	52280.01

III. DIPOLE MOMENTS

TABLE S6. Calculated dipole moments for non-symmetric molecules.

Molecule	Dipole moment [D]
MgH	1.343
CaH	2.516
^{1}MgCa	0.069
^{3}MgCa	1.603
Linear MgCC	4.510
Linear CaCC	7.421
Cyclic MgC_2	7.825
Cyclic CaC_2	11.041
MgCCH	1.594
CaCCH	2.644
^{1}MgCCCa	1.282
^{3}MgCCCa	1.274

IV. HARMONIC FREQUENCIES

All frequencies are given in cm^{-1}.

TABLE S7. Harmonic frequencies for M-X type molecules. Note the significantly lower vibrational frequencies for singlet species compared to the corresponding triplet, due to the qualitatively different nature of the bonding.

Molecule	ω
H_2	4403.47
MgH	1501.08
CaH	1352.82
$^1Mg_2$	47.28
$^3Mg_2$	233.28
$^1Ca_2$	59.96
$^3Ca_2$	162.11
^{1}MgCa	55.49
^{3}MgCa	205.67

TABLE S8. Harmonic frequencies for MC_2 type molecules.

Molecule	ω_1	ω_2	ω_3[a]
Linear MgCC	1942.99	493.85	96.38
Linear CaCC	1726.37	422.46	–[b]
Cyclic MgC_2	1741.47	586.80	438.47
Cyclic CaC_2	1778.20	549.27	425.53

[a] ω_3 corresponds to the degenerate bend in the linear species, following Woon.[1]

[b] This calculated potential well of linear CaCC is shallow and this vibrational frequency tended to imaginary values.[2]

TABLE S9. Harmonic frequencies for M-CC-X type molecules

Molecule	ω_1	ω_2	ω_3	ω_4	ω_5
HCCH	3508.37	2013.41	3416.05	606.28	747.65
MgCCH	3430.97	2039.85	500.34	678.42	150.53
CaCCH	3458.34	1984.71	399.86	676.69	116.90
^{1}MgCCMg^a	2081.61	362.72	623.93	214.02	81.27
^{3}MgCCMg^a	2082.89	363.14	624.88	207.44	81.25
^{1}CaCCCa^a	2022.63	250.79	507.54	107.14	51.61
^{3}CaCCCa^a	2022.66	250.90	507.74	106.32	51.02
^{1}CaCCMg^a	2053.09	578.74	295.65	175.38	63.28
^{3}CaCCMg^a	2053.40	579.33	295.81	172.55	62.88
^{3}MgCCMg^b	2021.17	357.77	620.92	180.16	67.70
^{3}CaCCCa^b	1958.38	247.58	489.55	97.47	40.38
^{3}CaCCMg^b	2053.79	569.35	287.84	151.29	49.63

^{a}Harmonic frequencies calculated with EOM-DIP-CCSD/pCVQZ for singlet cases with significant multi-reference character and in the analogous triplet states for consistency. EOM vibrational calculations for Ca-bearing molecules were carried out without an ECP. The CaCCCa dianion reference suffers from orbital instability in the one σ_u^+ displaced point that only affects ω_3.

^{b} CCSD(T) values for triplet species for comparison.

V. KITE SHAPED Mg_2C_2

TABLE S10. Structure of kite-shaped Mg_2C_2 stationary point with D_{2h} symmetry

	R(MC) [Å]	∠(CMC) ∘	∠(MCM) ∘
Geometry	2.1794	33.67	146.32

VI. COMPUTATIONAL VS. EXPERIMENTAL COMPARISON

TABLE S11. Comparison of calculated structural information vs. experimental literature values for linear species. Bond lengths in Å and rotational constants in cm^{-1}, equilibrium (r_e) and semi-experimental (SE) values as marked. The errors are all below 3%.

Molecule	Property	Computational	Experimental[3,4]	Error [%]
H_2[3]	r_e(H-H)	0.7419	0.7414[3]	0.06
	B_e	60.7239	60.853	0.21
Mg_2[3]	r_e(Mg-Mg)	3.9464	3.8905	1.44
	B_e	0.090254	0.09287	2.82
Ca_2[3]	r_e(Ca-Ca)	4.3421	4.2773	1.51
	B_e	0.044747	0.046113	2.96
MgH[3]	r_e(Mg-H)	1.7295	1.7297	0.01
	B_e	5.824877	5.82552	0.01
CaH[3]	r_e(Ca-H)	2.0037	2.0025	0.06
	B_e	4.27130	4.2766	0.12
MgCCH[4]	r_e^{SE}(M-C)	2.0393	2.0369	0.12
	r_e^{SE}(CC)	1.2228	1.2247	0.15
	r_e^{SE}(C-H)	1.0650	1.0647	0.02
	B_e^{SE}	4947.39012	4965.3354	0.36
CaCCH[4]	r_e^{SE}(M-C)	2.3156	2.3106	0.22
	r_e^{SE}(CC)	1.2282	1.2282	0.002
	r_e^{SE}(C-H)	1.0656	1.0655	0.01
	B_e^{SE}	3369.7042	3396.491	0.79

VII. COMPUTATIONAL COMPARISONS

TABLE S12. Singlet-triplet gaps (eV) for bimetallic species obtained by different computational methods. The EOM-DIP values are adiabatic, and the EOM-SF[5] are vertical. Regular CC methods are not expected to be correct for the singlet states, and so the (adiabatic) values are given here as a cautionary note.

Molecule	EOM-DIP-CCSD/pCVQZ	EOM-SF-CCSD/aug-cc-pwCVTZ(-PP)[5]	CCSD(T)/pCVQZ
MgCCMg	0.0056	0.016	0.291
CaCCCa	−0.0001	0.003	0.176
CaCCMg	0.002	0.008	0.0007

REFERENCES

[1] David Woon. Ab Initio Characterization of MgCCH, $MgCCH^+$, and MgC_2 and Pathways to Their Formation in the Interstellar Medium. *The Astrophysical Journal*, 456:602–10, 1996.

[2] Guosen Wang, Xia Huang, Changmin Guo, Hong Zhang, Chuanyu Zhang, and Xinlu Cheng. Dynamics Study of the CaC $(X^3\Sigma^-)$+C$(^3P_g) \rightarrow$ Ca+C_2 $(\Sigma\nu)$ Reaction: Based on a Full-Dimensional Neural Network Potential Energy Surface of CaC_2. *The Journal of Physical Chemistry A*, 129(8):2024–2032, 2025. PMID: 39951001.

[3] K. P. Huber and G. Herzberg. *Constants of Diatomic Molecules*, pages 8–689. Springer US, Boston, MA, 1979.

[4] P. Bryan Changala, Nadav Genossar-Dan, Ella Brudner, Tomer Gur, Joshua H. Baraban, and Michael C. McCarthy. Structural and Electronic Trends of Optical Cycling Centers in Polyatomic Molecules Revealed by Microwave Spectroscopy of MgCCH, CaCCH, and SrCCH. *Proceedings of the National Academy of Sciences*, 120(28):e2303586120, 2023.

[5] Maxim V. Ivanov, Sahil Gulania, and Anna I. Krylov. Two Cycling Centers in One Molecule: Communication by Through-Bond Interactions and Entanglement of the Unpaired Electrons. *The Journal of Physical Chemistry Letters*, 11(4):1297–1304, 2020.
